# A new risk model comprising genes highly correlated with CD133 identifies different tumor-immune microenvironment subtypes impacting prognosis in hepatocellular carcinoma

**DOI:** 10.18632/aging.103409

**Published:** 2020-06-20

**Authors:** Huajian Yu, Xiaoqiang Zhu, Hechun Lin, Hongyu Pan, Fangyu Zhao, Miaoxin Zhu, Lei Sun, Wenjun Chai, Ming Yao, Mingxia Yan

**Affiliations:** 1 State Key Laboratory of Oncogenes and Related Genes, Shanghai Cancer Institute, Renji Hospital, Shanghai Jiao Tong University School of Medicine, Shanghai, China; 2 School of Biomedical Sciences, Li Ka Shing Faculty of Medicine, The University of Hong Kong, Pok Fu Lam, Hong Kong SAR, China; 3 Fudan University Shanghai Cancer Center, Shanghai Medical College, Fudan University, Shanghai, China

**Keywords:** hepatocellular carcinoma, CD133, tumor-immune microenvironment, tumor immunology, prognostic model

## Abstract

The existence of cancer stem cells (CSCs), marked by CD133, is the primary cause of death in hepatocellular carcinoma (HCC). Here, we generated a new risk model comprising the signatures of four genes highly correlated with CD133 (CD133_(hi)_) that help improve survival in HCC. Three datasets were used to identify the differential CD133_(hi)_ genes by comparing sorted CD133^+^ liver CSCs and CD133^-^ differentiated counterparts. Univariate analysis was used to screen significantly differential CD133_(hi)_ genes associated with overall survival in the training dataset, which were used for risk model construction. High-risk patients were strongly associated with poor survival by Kaplan-Meier survival analysis in both the training and validation datasets. Clinical stratification analyses further demonstrated that the risk factors acted as independent factors and that high-risk patients were characterized by more aggressive cancer features. Functional enrichment analyses performed by gene set enrichment analysis (GSEA) and the Database for Annotation, Visualization and Integrated Discovery (DAVID) revealed that high-risk patients showed the disturbance of immune hepatic homeostasis involving aberrant immune cells, including macrophages and T and B cells, and an abnormal inflammatory response including the IL6/Jak/STAT3 pathway and TNF signaling pathway. In conclusion, our constructed CD133_(hi)_ gene risk model provides a resource for understanding the role of CD133^+^ CSCs in the progression of HCC in terms of tumor-immune interactions.

## INTRODUCTION

Hepatocellular carcinoma (HCC) is a very deadly and aggressive disease and represents the second leading cause of cancer-related mortality worldwide [1]. Although the treatment of HCC has greatly improved over the last decade, the disease remains a major health concern due to its resistance to chemotherapy, its high rate of recurrence, and our limited understanding of the mechanisms underlying the initiation and progression of the disease.

Cancer stem cells (CSCs) are considered to represent a small population of cancer cells that are responsible for tumor relapse, metastasis, drug resistance and evasion of the immune system [2, 3]. CD133 has been shown to be a marker of a liver CSC subset, and CD133^+^ HCC cells are well known for their role in frequent relapse, drug resistance, tumor initiation, sustained self-renewal, differentiation and phenocopying of the original tumor [4–6]. Moreover, compelling evidence has emerged in support of the intimate relationship between the tumorigenicity of CD133^+^ liver tumor-initiating cells and generally worse overall survival [5].

The HCC microenvironment is characterized by the presence of endothelial cells, hepatic stellate cells, tumor-associated macrophages (TAMs), regulatory T cells, cancer-associated fibroblasts, and CSCs mixed within an excessive accumulation of extracellular matrix (ECM). This microenvironment acts as a fertile environment to grow cancer seeds [7]. The disturbance of immune homeostasis among tumor cells, CSCs (CSCs) and the associated stroma is frequently associated with prolonged and sustained tumor dissemination in HCC [8]. The development of effective treatments for HCC has been hindered by the enhanced expression of the CSCs marker CD133, which has been reported to be aberrantly regulated by abnormal inflammatory factors, such as IL-6/STAT3, and effector immune cells. Moreover, a previous report demonstrated that therapeutic components that target TME by downregulating the expression of CSC markers, including CD133, showed evident antitumor effects [9].

It is of paramount significance to construct a robust and reliable prognostic molecular model that can provide more knowledge about the infiltrative nature of CD133^+^ liver CSC cells in HCC to find a new method of achieving early diagnosis and improving the overall survival of HCC patients. However, few of the biomarkers correlated with CD133 have been applied in clinical practice. Since CD133-positive cells, unlike CD133-negative cells, display more aggressiveness in HCC, our present study aims to generate a new genetic risk model comprised of genes highly correlated with CD133 (CD133_(hi)_) to gain better insights into the relationship between CD133_(hi)_ molecular markers and the prognosis of patients with HCC. We hope to find promising strategies for HCC treatment related to changing the tumor microenvironment (TME), especially the TME of CD133^+^ CSCs, which may provide a synergistic effect. This work is hence of clinical importance for identifying ways to advance immunotherapy research.

## RESULTS

### Enrichment pattern of individual liver CD133 markers

**Figure 1:**
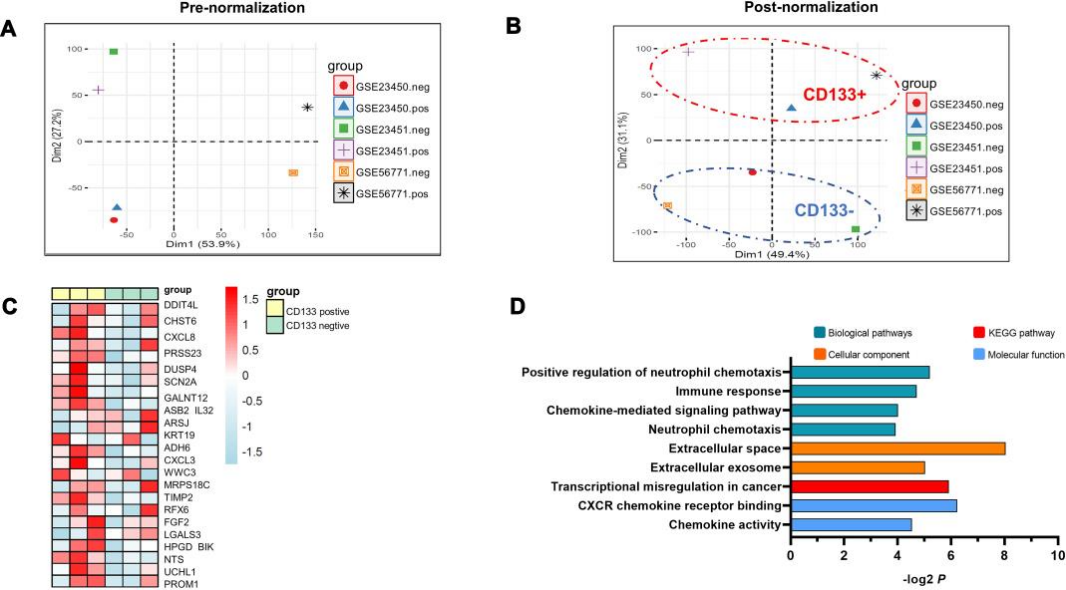
**PCA analysis of three RNA sequencing datasets compared to the differential gene expression between CD133^+^ liver CSCs and CD133^-^ differentiated counterparts.** (**A**) Data representing the clustering information of independent samples. The analysis was based on the expression of all genes for each individual sample. (**B**) Data representing the clustering information of independent samples after removing batch effects. The ellipses indicate group dispersion/variability from the centroid. CD133-positive groups are shown in the red ellipse, and CD133-negative groups are displayed in the blue ellipse. Two main components (PCA1/PCA2) of PCA were applied to the normalized differences to find the largest correlated variables. (**C**) Hierarchical cluster heatmap analysis of gene expression profiles in CD133^+^ liver CSCs vs. CD133^-^ liver CSCs. Each cell in the matrix represents a particular expression level, where the colors (white/green to pink/red) indicate lower to higher gene expression levels. The bars at the top of each column indicate the following: Yellow=CD133-positive group; green = CD133-negative group. At the bottom, from left to right, each column represents the sample name of the GEO dataset. (**D**) Pathway enrichment analysis of CD133^+^ liver CSCs. P values (–log2 transformed) are plotted for each enriched functional category. Abbreviations: PCA: principal component analysis; CD133^+^, CD133-positive groups; CD133^-^, CD133-negative groups.

We downloaded three RNA sequencing datasets in GEO (GSE23450, GSE23451, and GSE56771) that compare the differential gene expression between CD133^+^ liver CSCs and CD133^-^ differentiated counterparts. To systematically evaluate the differences, we combined all the cohorts together for analysis. PCA was performed by the “FactoMineR” and “Factoextra” packages in R software. As shown in Figure 1A, neither the CD133-negative group nor the CD133-positive group clustered together, revealing the presence of batch effects. Thus, we employed the “removeBatchEffect” program in the LIMMA package and finally obtained clean data by removing batch effects (Figure 1B). PCA also demonstrated a highly consistent trend in the CD133^+^ group and CD133^-^ counterpart obtained after combining both GEO datasets, in which the groups were separated into two major populations; PCA2 efficiently explained 31.1% of the variance (Figure 1B). These interesting results were consistent with previous reports of certain crucial factors at the genomic level in CD133^+^ liver CSCs that differed from those in CD133^-^ counterparts, which could explain the enhanced aggressive role of CD133^+^ cells in HCC [4].

### CD133^+^ liver CSCs showed different expression patterns compared with their CD133^-^ counterparts

To better understand the molecular changes underlying CD133^+^ liver CSC-driven HCC tumor progression, we further performed gene expression profiling by comparing the transcriptome profiles between the CD133^+^ liver CSC group and CD133^-^ counterpart. We next analyzed the differentially expressed genes with the criteria adj. P value < 0.05 and log2 |FC| >1. Differential expression patterns between the CD133^+^ liver CSC group and CD133^-^ counterpart were visualized using a hierarchical clustering heatmap (Figure 1C), which showed tremendously different gene expression patterns. Most of the deregulated genes (22 out of 24) were elevated.

### Pathway analysis revealed disturbance of the hepatic stroma in CD133^+^ liver CSCs

We performed pathway analysis using DAVID. As expected, the enriched KEGG pathways have been shown to be highly and significantly involved in transcriptional misregulation in cancer, suggesting that transcriptional modulation stimulating invasion and metastasis might be involved in the aggressive performance of CD133^+^ liver CSCs (Figure 1D). Moreover, we found that CD133^+^ liver CSCs caused multiple notable changes in the hepatic immune microenvironment. Of note, pathways involved in the immune response were dramatically enriched and revealed the evident infiltration of inflammatory cytokine genes, such as neutrophil chemotaxis and chemokine-mediated signaling pathways. Secreted proteins in the extracellular matrix were also deregulated significantly (Figure 1D), suggesting the disturbance of the hepatic stroma in HCC guided by CD133^+^ liver CSCs.

### Construction of a new prognostic scoring system based on the differentially expressed genes of tumorigenic liver CSCs

**Figure 2:**
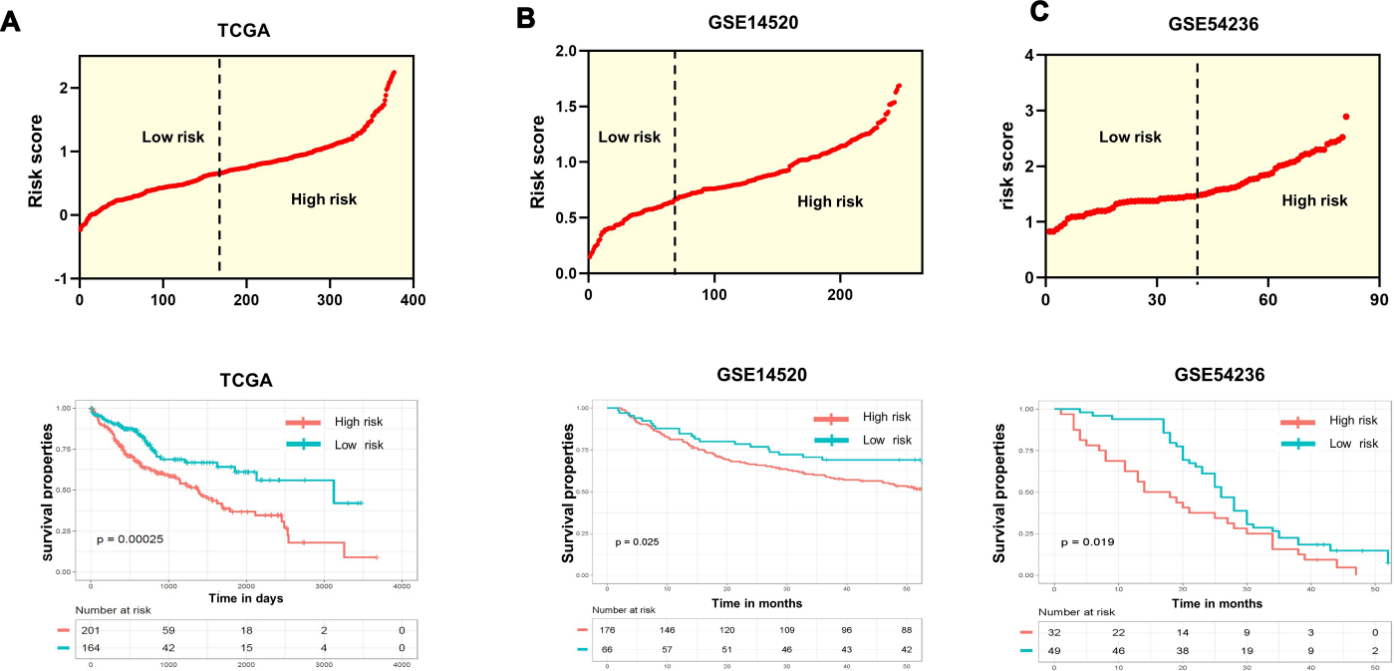
**Evaluation and validation of the survival predictions of the risk scoring system.** (**A**–**C**) Risk score distribution (upper) and Kaplan-Meier curves (lower) classifying patients into high- and low-risk groups by the new scoring system by comparing OS for patients in high- and low-risk groups in the training datasets and two test datasets. Patients with expression levels higher than the mean value are categorized into the high-risk group, while those with expression levels lower than the mean value are categorized into the low-risk group. P values were calculated by the log-rank test.

Because CD133^+^ CSCs have aggressive capabilities they serve as the primary cause of metastasis, therapy resistance and generally poor prognosis in HCC patients. In this study, we aimed to provide a practical tool by constructing a new risk model comprising CD133_(hi)_ that could predict patient prognosis. Thus, we conducted univariate survival analysis by applying Cox proportional hazard models of each gene to confirm the relevance between overall survival and the expression of CD133_(hi)_ genes in the training dataset (TCGA-LIHC). All the genes with significant P value (P value <0.05) were screened for the next step of model construction. Eventually, we identified 4 optimal genes (LGALS3, RFX6, ADH6, and UCHL1) significantly related to OS time in HCC patients (Supplementary Table 1). We then calculated the risk score of every patient (details are provided in the Methods section). The formula was as follows: risk score = 0.1376*(expression value of LGALS3) +0.1089*(expression value of RFX6) +(-0.0895)* (expression value of ADH6) +0.0742*(expression value of UCHL1). Patients with expression levels higher than the mean of all patients’ scores were divided into the high-risk group, while those with lower expression levels were separated into the low-risk group. Every patient with a new risk score is plotted in the upper portion of Figure 2A. Specifically, in the training dataset, a total of 201 patients whose risk scores were greater than the cutoff point were divided into the high-risk group, while the other 164 patients were assigned to the low-risk group, with risk scores below the cutoff value. Kaplan-Meier survival analysis was performed to compare the overall survival of the two groups of patients. The high-risk group was shown to be positively and significantly associated with poorer clinical outcomes than the low-risk group (p<0.0001) (Figure 2A; lower). Specifically, the five-year survival rate in the high-risk group of patients was 36.1% (95% CI, 28.3-48.6%), and that in the low-risk group of patients was 64.2% (95% CI, 54.1-74.2%), with a significant P value (P=0.00025).

### Validation of the prognostic model in an independent GEO dataset

To determine whether the effectiveness and predictive value of the prognostic model in predicting OS for patients with HCC was a common event, we extended our analysis to the other two datasets, GSE14520 and GSE54236. The same strategies were performed by separating the HCC patients into a low-risk group and a high-risk group according to the mean risk score (Figure 2B and 2C, upper). Consistent with the results in the training dataset, the high-risk patients had significantly shorter survival (Figure 2B and 2C, lower). Of note, in the GSE54236 dataset, after 18 months, more than half of the patients in the high-risk group were dead, while 75% of the patients in the low-risk group were alive.

### Independent prognostic factor analysis and clinical parameter stratification analysis

**Figure 3:**
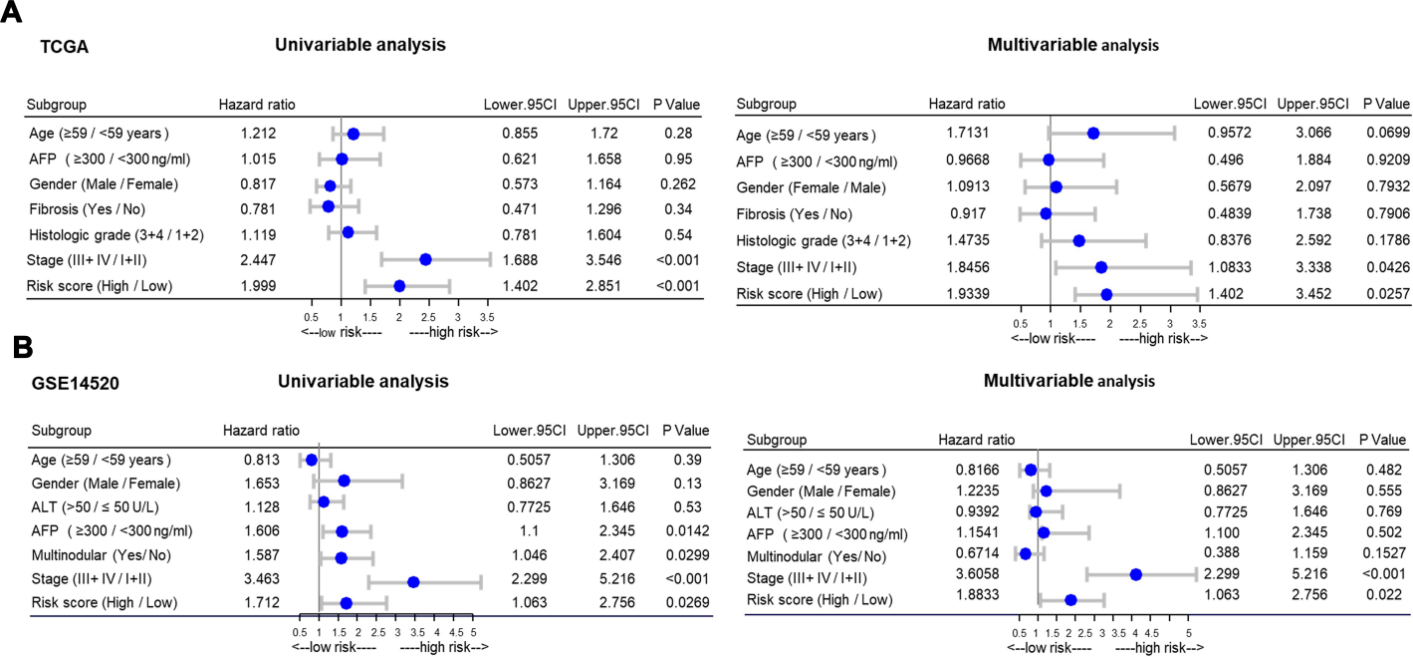
**Stratified analysis of overall survival in the TCGA and GSE14520 datasets by forestplot.** (**A**) Univariate (left) and multivariate (right) Cox regression analysis of the training group (TCGA). (**B**) Univariate (left) and multivariate (right) Cox regression analysis of the validation group (GSE14520); TCGA: The Cancer Genome Atlas; HR, hazard ratio; 95% CI, 95% confidence interval; AFP, α-fetoprotein; ALT, alanine aminotransferase; HCC, hepatocellular carcinoma.

**Figure 4:**
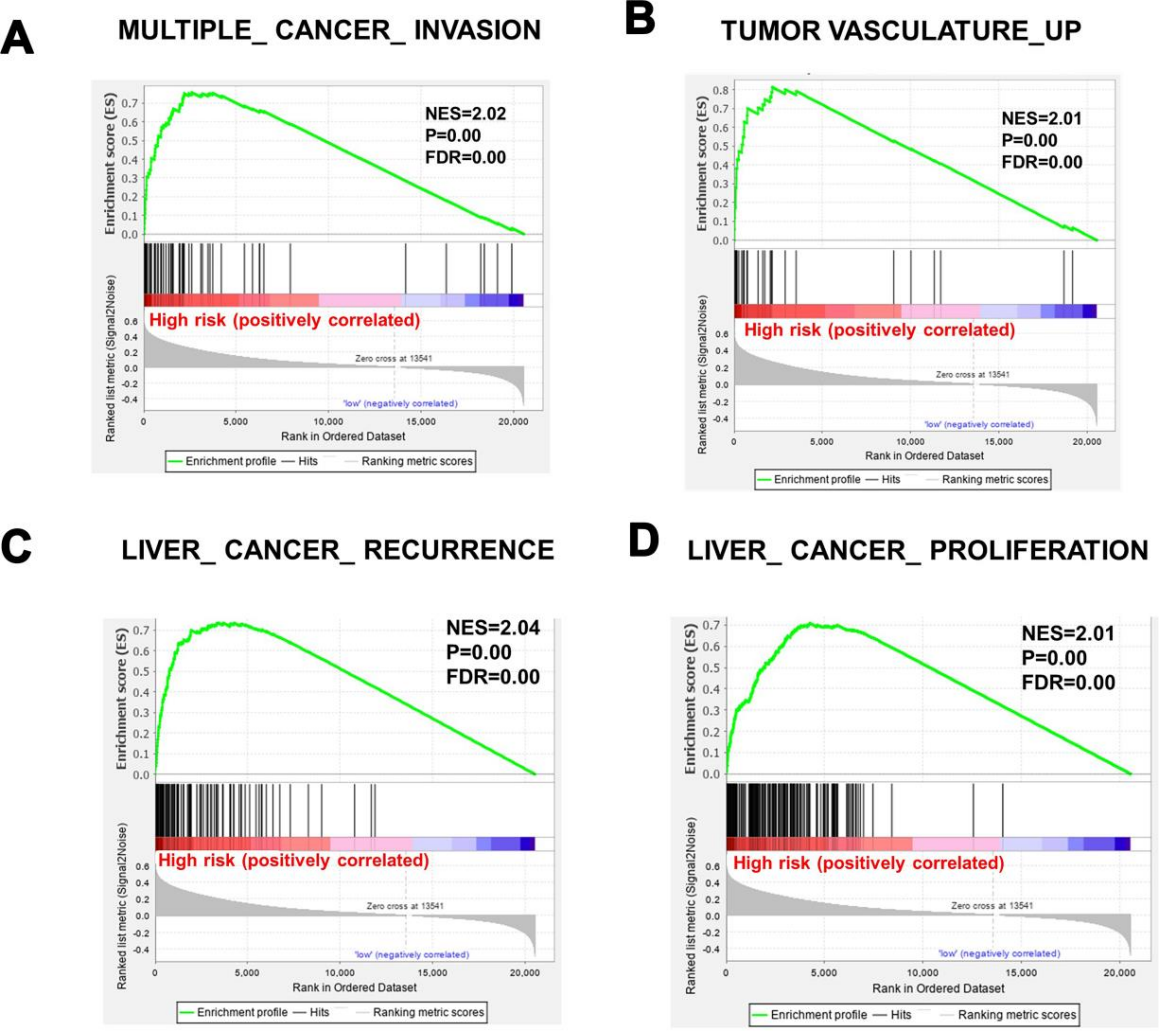
**Gene sets significantly enriched between high-risk patients and low-risk patients, determined using GSEA.** (**A**) MULTIPLE_CANCER_INVASION. (**B**) TUMOR VASCULATURE_UP. (**C**) LIVER_ CANCER_ RECURRENCE. (**D**) cell proliferation. NES, normalized enrichment score; FDR: false discovery rate.

To determine whether the high-risk group was correlated with aggressive clinical parameters, we performed clinical parameter stratification analysis. We stratified the patients into high- and low-risk groups according to the forecast model and correlated the signature with a series of clinical parameters in the two groups. Univariate and multivariate Cox regression analyses in both the training and validation groups indicated that our prognostic model was an independent prognostic factor for OS (Figure 3A and 3B). In addition, analysis of clinical parameters showed that high-risk patients were significantly associated with males (chi-square test, p=0.003), the late stage of HCC (stage III/IV, chi-square test, p=0.008) and lymph metastasis (chi-square test, p=0.032) in TCGA datasets (Table 1). In addition, we carried out GSEA analysis comparing the high-risk group and low-risk group to investigate key biological and cellular processes linked with poor prognosis. In the c2/curated gene set collection of the Molecular Signatures Database (MSigDB) of GSEA, the high-risk group displayed a certain number of deregulated pathways that have been reported to promote cancer aggressiveness. As shown, invasiveness signatures and genes related to tumor vasculature were among the leading enrichment gene sets of the high-risk groups (Figure 4A and 4B). The results also revealed notable enrichment in the recurrence of hepatitis B-related (HBV) hepatocellular carcinoma (HCC) (Figure 4C), suggesting that high-risk group patients were positively correlated with recurrence-free survival (Figure 4C, Supplementary Table 2). Moreover, multiple genes in the ‘proliferation’ subclass of HCC were evidently upregulated (Figure 4D). All of these genes are well-known deadly factors related to poor cancer prognosis.

**Table 1:** Summary of patient demographics and clinicopathologic characteristics associated with HCC in our study.

**Clinical Features**	**No. of cases**	**Low risk**	**High risk**	**P value**
Gender				
Female	121	51 (42.1%)	70 (57.9%)	0.003**
Male	250	147 (58.8%)	103 (41.2%)	
Age				
≤59	170	85 (50.0%)	85 (50.0%)	0.232
>59	201	113 (56.2%)	88 (43.8%)	
Tumor size				
<5 cm	45	3 (6.7%)	42 (93.3%)	0.796
≥5 cm	20	1 (5.0%)	19 (95.0%)	
AFP Elevation				
No	240	134 (55.8%)	106 (44.2%)	0.198
Yes	131	64 (48.9%)	67 (51.1%)	
TNM				
Stage I/II	257	150 (58.4%)	107 (41.6%)	0.008**
Stage III/IV	90	38 (42.2%)	52 (57.8%)	
Differentiation				
Well/Moderate	232	131 (56.5%)	101 (43.5%)	0.108
Poor	134	64 (47.8%)	70 (52.2%)	
Cirrhosis				
No	74	42 (56.8%)	32 (43.2%)	0.318
Yes	138	88 (63.8%)	50 (36.2%)	
Relapse				
No	229	122 (53.3%)	107 (46.7%)	0.963
Yes	142	76 (53.5%)	66 (46.5%)	
Metastasis				0.279
No	266	140 (52.6%)	126 (47.4%)	
Yes	4	1 (25.0%)	3 (75.0%)	
Lymph node metastasis				
No	252	136 (54%)	116 (46.0%)	0.032*
Yes	4	0 (0%)	4 (100%)	

Abbreviations: AFP, α-fetoprotein; P < 0.05 (*), P < 0.01 (**)

### Aggressive tumorigenesis properties were characterized by the disturbance of immune hepatic homeostasis in the high-risk patient group

**Figure 5:**
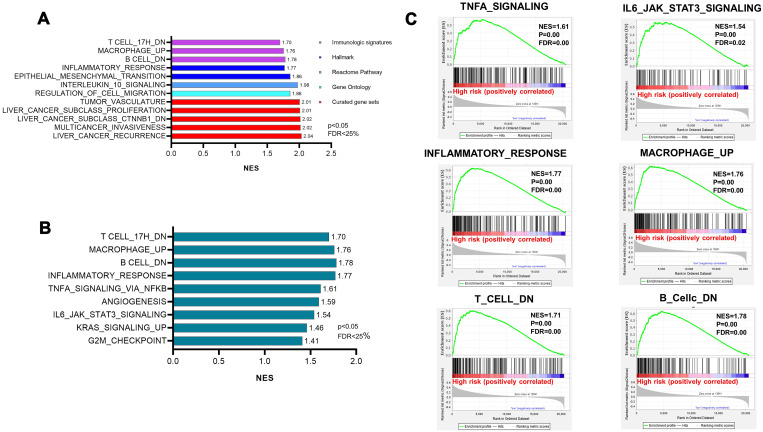
**Pathway enrichment showing the disturbance of immune hepatic homeostasis.** (**A**) The top gene sets of the hallmark gene sets, curated gene sets, Gene Ontology gene sets, and immunologic signature gene sets. NES>1.5 and FDR<0.25 were used as criteria. (**B**) The enriched pathways in the hepatic immune microenvironment generated from hallmark gene sets. (**C**) Highly enriched pathways related to inflammatory responses and the disturbance of immune cells.

Next, we interrogated changes in specific pathways based on TCGA to determine the risk factors behind the identified high-risk patients in our study. We performed GSEA analysis of the collection of Hallmark gene sets, Curated gene sets, Gene Ontology gene sets, and immunologic signature gene sets. The results of GSEA analysis also suggested a mechanism explaining why the high-risk group was related to poor prognosis. The top gene sets in each category according to NES, P value, and FDR are shown in Figure 5A. After interpreting our data, we found that epithelial-mesenchymal transition (EMT) was highly enriched in the high-risk group, and the NES value was 1.86, with a significant P value [Figure 5B]. Moreover, several notably enriched pathways in the hepatic immune microenvironment drew our attention. Of note, the upregulation of inflammatory responses included pathways connected with the IL6/Jak/STAT3 pathway and TNF signaling pathway (Figure 5C). Moreover, we observed the disturbance of immune cells, including B cells, T cells and macrophages (Figure 5C). The results showed that genes were downregulated in B lymphocytes and T cells in the high-risk groups and were upregulated in bone marrow-derived macrophages. These results suggest that the aggressive tumorigenesis properties of CD133^+^ CSCs are characterized by the disturbance of immune hepatic homeostasis in high-risk patient groups.

### An aberrant immune tumor microenvironment impacts prognosis in patients in the high-risk group

**Figure 6:**
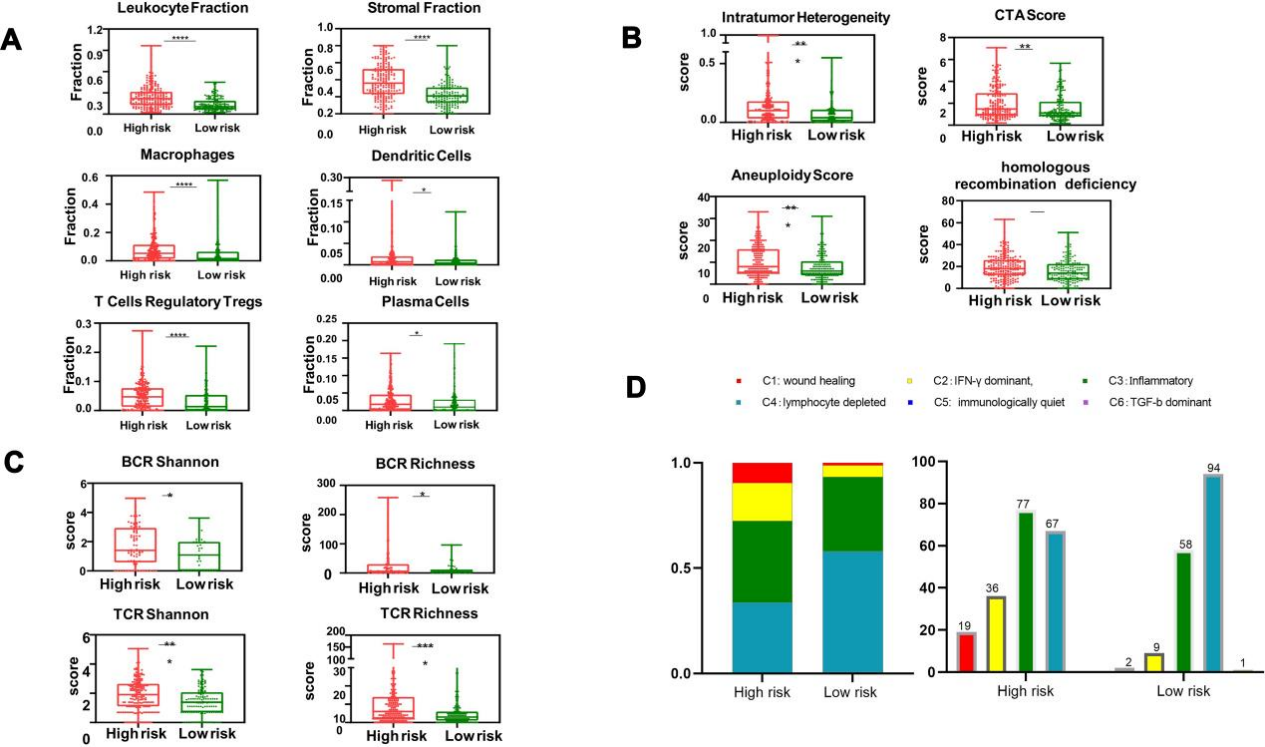
**Box and whisker chart showing the aberrant elevation of the tumor immune infiltrate in high-risk patients.** (**A**) The proportion of major classes of immune cells. (**B**) BCR (top) and TCR (bottom) diversity measured by the Shannon entropy and species richness. (**C**) Four key immune expression signature scores. (**D**) Number of patients and distribution of immune subtypes in the high-risk and low-risk groups. The text above the bars shows the specific number of patients in each immune subtype. The bar width reflects the number of tumor samples; the six immune subtypes include C1 (wound healing), C2 (IFN-γ dominant), C3 (inflammatory), C4 (lymphocyte depleted), C5 (immunologically quiet) and C6 (TGF-b dominant); values from min to max are plotted.

To evaluate whether tumor-extrinsic features can predict outcomes, we further focused on the immune response in patients in the high-risk group. We performed an extensive immunogenomic analysis by using the data generated by Thorsson et al*.* [10] to characterize the immune tumor microenvironment by comparing the patients in the high-risk and low-risk groups. Specifically, we found elevated leukocyte and immune cell fractions, including lymphocytes, dendritic cells, and macrophages (Figure 6A). Additionally, we measured the diversity of TCR and BCR through Shannon entropy and species richness (Figure 6B), which showed more diversity in high-risk patients than in lower-risk patients, indicating that high-risk patients might have more antigen-specific TCR and BCR repertoires to detect invading pathogens. In addition, patients in the high-risk group showed higher levels of aneuploidy, homologous recombination deficiency (HRD), and intratumor heterogeneity (Figure 6C), suggesting that the above substantial immune infiltrate might increase the probability of chromosomal instability, defective DNA repair and spatially heterogeneous tumor structures, promoting neoplastic transformation and functional development into distinct cell populations. Furthermore, according to the intratumoral immune states and immune subtypes (C1-C6) mentioned by Thorsson et al*.*, we found substantial variation in the proportion of immune subtypes in these two groups (Figure 6D). As illustrated, patients with HCC were rich in C3 and C4. A higher percentage of high-risk-group patients than low-risk-group patients were rich in C1, which has been reported to have less favorable outcomes [10].

## DISCUSSION

HCC is a deadly and silently progressing malignancy, ranking second in mortality among all human malignancies worldwide [1] Currently available biomarkers for this aggressive cancer are not sufficiently specific and sensitive to meet all clinical needs. A number of studies have reported the crucial role of CSCs (CSCs) in the progression of HCC in patients with advanced HCC. CSCs exhibit self-renewal ability and multilineage differentiation capabilities, enabling tumor cell metastasis invasion and resistance to chemotherapy, thus causing a high rate of death [11]. Moreover, CSCs in HCC have been reported to be characterized by the CD133 phenotype [4]. Patients who have a higher percentage of CD133^+^ liver tumor-initiating cells are related to poorer survival [6]. Therefore, there is an imminent and pressing need for strategies that can specifically target critical factors to improve the therapeutic effect for HCC patients.

Owing to the heterogeneity and complexity of the etiology and clinical characteristics of HCC, it is urgent to find a practical combination of biomarkers with high sensitivity and specificity instead of searching for a single biomarker for early diagnosis and prognostication of HCC. Our current study focused on the aggressive phenotype of CD133^+^ cells. We initially exploited data available in three GEO datasets comparing the differential gene expression between sorted CD133^+^ liver CSCs and CD133^-^ differentiated counterparts. Due to systematic nonbiological variation between groups of samples, we found batch effects in these three datasets. PCA is a well-known technique for reducing the dimensionality of such datasets and can increase interpretability while at the same time minimizing information loss by reducing the dimensionality of a dataset [12]. The utilization of R software (FactoMineR and factoextra packages) enabled us to remove batch effects and obtain normalized clean data. Furthermore, we unveiled 24 differentially expressed genes with high correlations with CD133. Most of the deregulated genes (22 out of 24) were elevated, suggesting that some oncogenic genes or signaling pathways were prominently activated. Next, by verifying survival data in TCGA through the univariate Cox regression method, we identified four important proteins, including LGALS3 (galectin-3), RFX6 (regulatory factor X6), ADH6 (alcohol dehydrogenase 6), and UCHL1 (ubiquitin C-terminal hydrolase L1). LGALS3, RFX6 and UCHL1 play a risk role in HCC patients, and in contrast, the other protein (ADH6) acts as a protective factor for HCC. LGALS3 is a member of the β-galactosidase-binding lectin family, mediating the proliferation, differentiation, and angiogenesis of tumor cells to promote cancer progression via endogenous and secretion mechanisms [13, 14]. Both endogenous and secreted LGALS3 were upregulated in HCC tumor tissue and serum samples [15, 16], which was highly correlated with poor survival. Moreover, *in vivo* studies demonstrated that LGALS3 deficiency was significantly associated with a smaller tumor burden, less invasive characteristics, reduced proliferation and an increased apoptosis rate [13, 16], while rLGALS3 showed the opposite effects [17, 18]. Alcohol dehydrogenase 6 (ADH6) is a liver-specific secreted protein belonging to the alcohol dehydrogenase family that encodes class V alcohol dehydrogenase, which controls retinol metabolism. ADH6 has been reported to be a novel circulating biomarker and may be involved in HCC pathogenesis [19]. Consistent with our data, ADH6 exhibited fivefold decreased expression in HCC secretome analysis compared to normal samples [20]. Furthermore, ADH6 has been reported to be a hypermethylated-repressed gene with aberrant DNA methylation on its promoter region, which may be responsible for its downregulation in HCC [21]. Ubiquitin carboxyl-terminal hydrolase L1 (UCHL1) is a member of the ubiquitin carboxyl-terminal esterase family, which belongs to the deneddylation enzymes [22]. Inconsistent with our data, accumulating evidence has shown that UCHL1 is overactivated in HCC, exhibiting its role in driving aggressive characteristics in HCC, and is related to poor overall survival. Additionally, UCHL1 upregulation demonstrated its role in conferring drug resistance by promoting apoptosis [23]. The gene regulatory factor 6 (RFX6) is a transcription factor that is involved in cancer progression in various cancers [24]. However, to the best of our knowledge, this is the first report of its significance in HCC. In addition, RFX6 was found to regulate the number of pancreatic progenitors, suggesting its potential involvement in cancer stemness [25]. We further investigated the statistical significance of clinical parameters, and the results showed that this new risk scoring system could serve as a potential and independent predictor for OS and metastasis, particularly in male patients. Further, we also performed the time-dependent receiver operator characteristic curve (ROC) curves in both the training dataset and two validation datasets, which demonstrating the accuracy of this prognostic model for predicting OS in HCC patients (Supplementary Figure 3).

To determine the primary cause of poor survival and aggressive cancer features, we next investigated the deep mechanisms that are deregulated in high-risk group patients. In this study, interestingly, both the enrichment pathway associated with the differential genes between CD133^+^ liver CSCs and their counterparts and that associated with the differential genes between the high-risk group and the low-risk group revealed that pathways related to the TME were dramatically enriched, especially those related to immune cells, the main cellular components, and extracellular components such as the extracellular matrix. This finding strongly suggests that high-risk group patients might generate tumor-promoting stroma in the liver. It is widely recognized that the hepatic microenvironment and stemness properties play a pivotal role in triggering HCC initiation and development [26]. Tumor stroma influences the processes of hepatocarcinogenesis, epithelial-to-mesenchymal transition, invasion, and metastasis [27]. The tumors in the high-risk group patients were hallmarked by increased inflammation and aberrant activation of the EMT pathway, IL6/Jak/STAT3 pathway and TNF-α signaling pathway. Consistent with our data, a recent report suggested the constitutive activation of IL-6-mediated inflammatory programs in accelerating the transformation of CD133^+^ liver stem cells into metastatic CSCs [28]. In addition, a recent report revealed that TNF-α signaling promoted the self-renewal and metastasis phenotypes of HCC cells [29]. Furthermore, we also showed the increased hepatic infiltration of macrophages in the high-risk groups, accompanied by downregulated levels of B and T cells. Our findings support several other studies with regard to antitumor activity. For instance, Marta et al demonstrated that the ablation of B cells resulted in enhanced tumor growth and reduced local T cell activation [26]. On the other hand, Schneider et al recognized that T and B cells were critical for the suppression of HCC progression [30]. Another study performed by Nielson et al reported that tumor-infiltrating B cells with an atypical CD27−memory phenotype were correlated with better prognosis in ovarian cancer [31]. We believe that elevated inflammatory responses, especially increases in IL-6 and TNF-α signaling, as well as decreases in B and T cells, accounted for, at least in part, severe HCC development in the high-risk-group patients. According to a previous study [10], C1 is one of the immune subtypes that shows elevated expression of angiogenic genes and a high proliferation rate. A higher percentage of high-risk-group patients than low-risk-group patients was rich in C1, suggesting that the immune microenvironment promotes the proliferation and angiogenesis of tumors [10]. Additionally, we found more diverse TCR and BCR repertoires in high-risk patients than in low-risk patients; such diversity is critical for the recognition of pathogens and malignant cells and may increase the degree of clonal expansion and challenges of antitumor drug therapy [32, 33]. Moreover, the high-risk group exhibited significantly higher levels of aneuploidy, homologous recombination deficiency (HRD), and intratumor heterogeneity than the low-risk group, suggesting that these processes lead to tumor development through different routes. High-risk patients might develop carcinogenesis through chromosomal instability (CIN) [34], impairing the DNA repair pathway [35], which is often associated with poor prognosis and drug resistance in cancers. In addition, it would be interesting to see how this model behaves when predicting inflammatory conditions of the liver. Thus, we separated patients into the hepatitis group and the non-hepatitis group. Interestingly, we found that this model behaves well when considering inflammatory conditions of the liver. As shown in Supplementary Figure 2A and 2B), we found that the high-risk group was significantly related to poor prognosis in both groups, suggesting that this model is independent of inflammatory conditions of the liver.

The CD133 fraction is mainly expressed in endothelial cells, according to the latest discoveries by Nadim Aizarani et al [39], published in Nature. Their work successfully improved our knowledge of the cellular composition of the liver by performing single-cell RNA sequencing of ~10,000 cells from normal liver tissue from 9 human donors, providing an interactive human liver cell atlas. This atlas comprises all the main liver cell types, including hepatocytes, bile duct cells, liver sinusoidal endothelial cells (LSECs), macrovascular endothelial cells (MaVECs), hepatic stellate cells, Kupffer cells, and immune cells. (http://human-liver-cell-atlas.ie-freiburg.mpg.de/). Here, the epithelial cell marker EPCAM showed epithelial cell clusters (Supplementary Figure 1; right). The t-SNE maps clearly demonstrated that CD133 showed a consistently high expression level in epithelial cells (Supplementary Figure 1; left). In addition, as proof of concept, CD133 is a marker of CSCs in HCC, and we found that CD133 showed relatively high expression levels in cluster 7. As mentioned in the scientific work by Nadim Aizarani et al, cluster 7 is the MUC6^high^ population, which has been proposed to be the origin of EPCAM+ hepatic stem cells.

In conclusion, we identified a new risk model comprising genes highly correlated with CD133 that revealed the disturbance of immune hepatic homeostasis in HCC, especially the late stage of HCC in male patients, which can forecast survival in HCC, with higher risk scores indicating poor prognosis. Patients, especially males, who are in the high-risk group may have protumorigenic stromal interactions in the liver, thereby facilitating tumor growth and metastasis. This work provides a resource for understanding the impact of CD133^+^ CSCs (CSCs) on the progression of HCC in terms of tumor-immune interactions and has potential therapeutic and prognostic implications for identifying ways to advance immunotherapy research.

## MATERIALS AND METHODS

### Data collection and preprocessing

Three genomic profiling datasets (GSE23450, GSE23451, and GSE56771) and a validation dataset (GSE54236) were retrieved from the GEO database (https://www.ncbi.nlm.nih.gov/geo/). GSE23450 and GSE23451 contain CD133^+^ and CD133^-^ subpopulations sorted from Huh7 and PLC8024 HCC cells. The GSE56771 data were uploaded by another group and include information on CD133^+^ and CD133^-^ cells sorted from Huh7 cells. The training dataset was downloaded from the database of the Liver Hepatocellular Carcinoma (LIHC) project (https://portal.gdc.cancer.gov/projects/TCGA-LIHC). A total of 365 HCC samples were included in the analysis of the training group. Eighty-one tumor samples and 242 tumor specimens were included in the validation datasets GSE54236 and GSE14520, respectively. We performed principal component analysis (PCA) by reducing the dimensionality of the datasets [12]. Next, we used the well-known FactoMineR and factoextra packages in R software to reduce the dimension of the feature space and to visualize the existence of batch effects [36]. Later, the batch effects were removed by using the removeBatchEffect program in the LIMMA package.

### Screening of differentially expressed CD133_(hi)_ genes

The CEL files and probe annotation files in the three genomics profiling datasets were downloaded and combined for analysis through genome-wide microarray analysis. Next, we performed gene differential expression analysis using the LIMMA package (Version 3.36.2). The Affy package was used to normalize the raw data by performing average background correction, quantile normalization and calculation of expression, after which a linear model was fitted, and empirical Bayes statistics were computed [37]. Clustering analysis of up- and downregulated differentially expressed genes was performed using the Pheatmap package in R statistical software.

### Construction of the prognostic model

The Cox proportional hazards model is essentially a regression model that is commonly used in statistical cancer research to investigate the association between the survival time of patients and other predictor variables. Univariate survival analysis of the individual differentially expressed CD133_(hi)_ genes was performed by the Survival package in R. The coefficients, P values and hazard ratios (HRs) with 95% confidence intervals (CIs) of each gene were generated in the risk scoring system based on the expression of each sample in R studio at the same time. We then calculated the risk score of every patient. The prognostic model was constructed based on a linear combination of expression levels weighted by regression coefficients. A P value<0.05 was considered a significant difference.

### Confirmation and evaluation of the power of the new risk scoring system

Based on the established model, we separated patients into high-risk groups and low-risk groups in both the training group and validation group according to the PI. Patients with expression levels higher than the mean value were categorized into the high-risk group, while those with expression levels lower than the mean value were categorized into the low-risk group. The survival curves of the two groups were plotted using Kaplan-Meier survival curves, and the difference was tested using the log-rank method.

### Functional and pathway enrichment analysis

Functional enrichment analyses of the differential CD133_(hi)_ genes, including Gene Ontology (GO) annotation analysis and Kyoto Encyclopedia of Genes and Genomes (KEGG) pathway enrichment analysis of DEGs, were carried out using DAVID [37]. Gene set enrichment analysis (GSEA) [38] using a preranked tool was carried out by the program JAVA (http://www.broadinstitute.org/gsea) based on the collection of gene sets in the Molecular Signatures Database (MsigDB). A normalized enrichment score (NES) was generated for each gene set to compare the analysis results across gene sets. A false discovery rate (FDR) <0.25 was considered as a well-established cutoff to determine enrichment terms. A gene set P value < 0.05 was considered statistically significant.

### Statistics

Statistical analysis was conducted using GraphPad Prism 8 software. Clinical parameters of the patients in the high-risk group and low-risk group were evaluated by the chi-squared test. Statistical significance was calculated by Student’s t-test, with P<0.05 considered significant.

## Supplementary Material

Supplementary Figures

Supplementary Tables

## References

[1] Siegel R, Naishadham D, Jemal A. Cancer statistics, 2013. CA Cancer J Clin. 2013; 63:11–30. 10.3322/caac.21166 23335087

[2] Pang RW, Poon RT. Cancer stem cell as a potential therapeutic target in hepatocellular carcinoma. Curr Cancer Drug Targets. 2012; 12:1081–94. 10.2174/156800912803987995 22873219

[3] Lee TK, Cheung VC, Lu P, Lau EY, Ma S, Tang KH, Tong M, Lo J, Ng IO. Blockade of CD47-mediated cathepsin s/protease-activated receptor 2 signaling provides a therapeutic target for hepatocellular carcinoma. Hepatology. 2014; 60:179–91. 10.1002/hep.27070 24523067

[4] Ma S, Chan KW, Hu L, Lee TK, Wo JY, Ng IO, Zheng BJ, Guan XY. Identification and characterization of tumorigenic liver cancer stem/progenitor cells. Gastroenterology. 2007; 132:2542–56. 10.1053/j.gastro.2007.04.025 17570225

[5] Ma S, Tang KH, Chan YP, Lee TK, Kwan PS, Castilho A, Ng I, Man K, Wong N, To KF, Zheng BJ, Lai PB, Lo CM, et al. miR-130b promotes CD133(+) liver tumor-initiating cell growth and self-renewal via tumor protein 53-induced nuclear protein 1. Cell Stem Cell. 2010; 7:694–707. 10.1016/j.stem.2010.11.010 21112564

[6] Tang KH, Ma S, Lee TK, Chan YP, Kwan PS, Tong CM, Ng IO, Man K, To KF, Lai PB, Lo CM, Guan XY, Chan KW. CD133(+) liver tumor-initiating cells promote tumor angiogenesis, growth, and self-renewal through neurotensin/interleukin-8/CXCL1 signaling. Hepatology. 2012; 55:807–20. 10.1002/hep.24739 21994122

[7] Hernandez-Gea V, Toffanin S, Friedman SL, Llovet JM. Role of the microenvironment in the pathogenesis and treatment of hepatocellular carcinoma. Gastroenterology. 2013; 144:512–27. 10.1053/j.gastro.2013.01.002 23313965 PMC3578068

[8] Hanahan D, Coussens LM. Accessories to the crime: functions of cells recruited to the tumor microenvironment. Cancer Cell. 2012; 21:309–22. 10.1016/j.ccr.2012.02.022 22439926

[9] Rodríguez MM, Fiore E, Bayo J, Atorrasagasti C, García M, Onorato A, Domínguez L, Malvicini M, Mazzolini G. 4Mu decreases CD47 expression on hepatic cancer stem cells and primes a potent antitumor T cell response induced by interleukin-12. Mol Ther. 2018; 26:2738–50. 10.1016/j.ymthe.2018.09.012 30301668 PMC6277513

[10] Thorsson V, Gibbs DL, Brown SD, Wolf D, Bortone DS, Ou Yang TH, Porta-Pardo E, Gao GF, Plaisier CL, Eddy JA, Ziv E, Culhane AC, Paull EO, et al, and Cancer Genome Atlas Research Network. The immune landscape of cancer. Immunity. 2018; 48:812–30.e14. 10.1016/j.immuni.2018.03.023 29628290 PMC5982584

[11] Ayob AZ, Ramasamy TS. Cancer stem cells as key drivers of tumour progression. J Biomed Sci. 2018; 25:20. 10.1186/s12929-018-0426-4 29506506 PMC5838954

[12] Jolliffe IT, Cadima J. Principal component analysis: a review and recent developments. Philos Trans A Math Phys Eng Sci. 2016; 374:20150202. 10.1098/rsta.2015.0202 26953178 PMC4792409

[13] Jiang SS, Weng DS, Wang QJ, Pan K, Zhang YJ, Li YQ, Li JJ, Zhao JJ, He J, Lv L, Pan QZ, Xia JC. Galectin-3 is associated with a poor prognosis in primary hepatocellular carcinoma. J Transl Med. 2014; 12:273. 10.1186/s12967-014-0273-3 25260879 PMC4179848

[14] Tummala KS, Brandt M, Teijeiro A, Graña O, Schwabe RF, Perna C, Djouder N. Hepatocellular carcinomas originate predominantly from hepatocytes and benign lesions from hepatic progenitor cells. Cell Rep. 2017; 19:584–600. 10.1016/j.celrep.2017.03.059 28423321 PMC5409928

[15] Eisa NH, Ebrahim MA, Ragab M, Eissa LA, El-Gayar AM. Galectin-3 and matrix metalloproteinase-9: perspective in management of hepatocellular carcinoma. J Oncol Pharm Pract. 2015; 21:323–30. 10.1177/1078155214532698 24769518

[16] Serizawa N, Tian J, Fukada H, Baghy K, Scott F, Chen X, Kiss Z, Olson K, Hsu D, Liu FT, Török NJ, Zhao B, Jiang JX. Galectin 3 regulates HCC cell invasion by RhoA and MLCK activation. Lab Invest. 2015; 95:1145–56. 10.1038/labinvest.2015.77 26146960 PMC4586310

[17] Wang M, Tian F, Ying W, Qian X. Quantitative proteomics reveal the anti-tumour mechanism of the carbohydrate recognition domain of galectin-3 in hepatocellular carcinoma. Sci Rep. 2017; 7:5189. 10.1038/s41598-017-05419-5 28701735 PMC5507876

[18] Lin L, Han MM, Wang F, Xu LL, Yu HX, Yang PY. CXCR7 stimulates MAPK signaling to regulate hepatocellular carcinoma progression. Cell Death Dis. 2014; 5:e1488. 10.1038/cddis.2014.392 25341042 PMC4649507

[19] Awan FM, Naz A, Obaid A, Ali A, Ahmad J, Anjum S, Janjua HA. Identification of circulating biomarker candidates for hepatocellular carcinoma (HCC): an integrated prioritization approach. PLoS One. 2015; 10:e0138913. 10.1371/journal.pone.0138913 26414287 PMC4586137

[20] Ding W, Qiu Q, Liu G, Liu J, Mao R, Lin B. Metadata checklist: identification of CHI3L1 and MASP2 as a biomarker pair for liver cancer through integrative secretome and transcriptome analysis. OMICS. 2014; 18:658–61. 10.1089/omi.2014.0090 25133456 PMC4175428

[21] Udali S, Guarini P, Ruzzenente A, Ferrarini A, Guglielmi A, Lotto V, Tononi P, Pattini P, Moruzzi S, Campagnaro T, Conci S, Olivieri O, Corrocher R, et al. DNA methylation and gene expression profiles show novel regulatory pathways in hepatocellular carcinoma. Clin Epigenetics. 2015; 7:43. 10.1186/s13148-015-0077-1 25945129 PMC4419480

[22] Yu J, Huang WL, Xu QG, Zhang L, Sun SH, Zhou WP, Yang F. Overactivated neddylation pathway in human hepatocellular carcinoma. Cancer Med. 2018; 7:3363–72. 10.1002/cam4.1578 29846044 PMC6051160

[23] Yang G, Fan G, Zhang T, Ma K, Huang J, Liu M, Teng X, Xu K, Fan P, Cheng D. Upregulation of ubiquitin carboxyl-terminal hydrolase L1 (UCHL1) mediates the reversal effect of verapamil on chemo-resistance to adriamycin of hepatocellular carcinoma. Med Sci Monit. 2018; 24:2072–82. 10.12659/msm.908925 29627846 PMC5909418

[24] Huang Q, Whitington T, Gao P, Lindberg JF, Yang Y, Sun J, Väisänen MR, Szulkin R, Annala M, Yan J, Egevad LA, Zhang K, Lin R, et al. A prostate cancer susceptibility allele at 6q22 increases RFX6 expression by modulating HOXB13 chromatin binding. Nat Genet. 2014; 46:126–35. 10.1038/ng.2862 24390282

[25] Zhu Z, Li QV, Lee K, Rosen BP, González F, Soh CL, Huangfu D. Genome editing of lineage determinants in human pluripotent stem cells reveals mechanisms of pancreatic development and diabetes. Cell Stem Cell. 2016; 18:755–68. 10.1016/j.stem.2016.03.015 27133796 PMC4892994

[26] Garnelo M, Tan A, Her Z, Yeong J, Lim CJ, Chen J, Lim KH, Weber A, Chow P, Chung A, Ooi LL, Toh HC, Heikenwalder M, et al. Interaction between tumour-infiltrating B cells and T cells controls the progression of hepatocellular carcinoma. Gut. 2017; 66:342–51. 10.1136/gutjnl-2015-310814 26669617 PMC5284473

[27] Novikova MV, Khromova NV, Kopnin PB. Components of the hepatocellular carcinoma microenvironment and their role in tumor progression. Biochemistry (Mosc). 2017; 82:861–73. 10.1134/S0006297917080016 28941454

[28] Mitra A, Yan J, Xia X, Zhou S, Chen J, Mishra L, Li S. IL6-mediated inflammatory loop reprograms normal to epithelial-mesenchymal transition^+^ metastatic cancer stem cells in preneoplastic liver of transforming growth factor beta-deficient β2-spectrin^+/-^ mice. Hepatology. 2017; 65:1222–36. 10.1002/hep.28951 27863449 PMC5360560

[29] Zhao B, Wang Y, Tan X, Ke K, Zheng X, Wang F, Lan S, Liao N, Cai Z, Shi Y, Zheng Y, Lai Y, Wang L, et al. Inflammatory micro-environment contributes to stemness properties and metastatic potential of HCC via the NF-κB/miR-497/SALL4 axis. Mol Ther Oncolytics. 2019; 15:79–90. 10.1016/j.omto.2019.08.009 31650028 PMC6804787

[30] Schneider C, Teufel A, Yevsa T, Staib F, Hohmeyer A, Walenda G, Zimmermann HW, Vucur M, Huss S, Gassler N, Wasmuth HE, Lira SA, Zender L, et al. Adaptive immunity suppresses formation and progression of diethylnitrosamine-induced liver cancer. Gut. 2012; 61:1733–43. 10.1136/gutjnl-2011-301116 22267597 PMC4533880

[31] Nielsen JS, Sahota RA, Milne K, Kost SE, Nesslinger NJ, Watson PH, Nelson BH. CD20+ tumor-infiltrating lymphocytes have an atypical CD27- memory phenotype and together with CD8+ T cells promote favorable prognosis in ovarian cancer. Clin Cancer Res. 2012; 18:3281–92. 10.1158/1078-0432.CCR-12-0234 22553348

[32] Zhu W, Germain C, Liu Z, Sebastian Y, Devi P, Knockaert S, Brohawn P, Lehmann K, Damotte D, Validire P, Yao Y, Valge-Archer V, Hammond SA, et al. A high density of tertiary lymphoid structure B cells in lung tumors is associated with increased CD4^+^ T cell receptor repertoire clonality. Oncoimmunology. 2015; 4:e1051922. 10.1080/2162402X.2015.1051922 26587322 PMC4635865

[33] Han Y, Li H, Guan Y, Huang J. Immune repertoire: a potential biomarker and therapeutic for hepatocellular carcinoma. Cancer Lett. 2016; 379:206–12. 10.1016/j.canlet.2015.06.022 26188280

[34] He Q, Au B, Kulkarni M, Shen Y, Lim KJ, Maimaiti J, Wong CK, Luijten MN, Chong HC, Lim EH, Rancati G, Sinha I, Fu Z, et al. Chromosomal instability-induced senescence potentiates cell non-autonomous tumourigenic effects. Oncogenesis. 2018; 7:62. 10.1038/s41389-018-0072-4 30108207 PMC6092349

[35] Hoppe MM, Sundar R, Tan DS, Jeyasekharan AD. Biomarkers for homologous recombination deficiency in cancer. J Natl Cancer Inst. 2018; 110:704–13. 10.1093/jnci/djy085 29788099

[36] Aizarani N, Saviano A, Sagar, Mailly L, Durand S, Herman JS, Pessaux P, Baumert TF, Grün D. A human liver cell atlas reveals heterogeneity and epithelial progenitors. Nature. 2019; 572:199–204. 10.1038/s41586-019-1373-2 31292543 PMC6687507

[37] Reese SE, Archer KJ, Therneau TM, Atkinson EJ, Vachon CM, de Andrade M, Kocher JP, Eckel-Passow JE. A new statistic for identifying batch effects in high-throughput genomic data that uses guided principal component analysis. Bioinformatics. 2013; 29:2877–83. 10.1093/bioinformatics/btt480 23958724 PMC3810845

[38] Yu H, Zhao F, Li J, Zhu K, Lin H, Pan Z, Zhu M, Yao M, Yan M. TBX2 identified as a potential predictor of bone metastasis in lung adenocarcinoma via integrated bioinformatics analyses and verification of functional assay. J Cancer. 2020; 11:388–402. 10.7150/jca.31636 31897234 PMC6930436

[39] Subramanian A, Tamayo P, Mootha VK, Mukherjee S, Ebert BL, Gillette MA, Paulovich A, Pomeroy SL, Golub TR, Lander ES, Mesirov JP. Gene set enrichment analysis: a knowledge-based approach for interpreting genome-wide expression profiles. Proc Natl Acad Sci USA. 2005; 102:15545–50. 10.1073/pnas.0506580102 16199517 PMC1239896

