# Supplementary material for: A new risk model comprising genes highly correlated with CD133 identifies different tumor-immune microenvironment subtypes impacting prognosis in hepatocellular carcinoma
Source: Aging (Albany NY). 2020 Jun 20;12(12):12234–50. doi: 10.18632/aging.103409 (PMC7343494; doi:10.18632/aging.103409)
Supplement: Supplementary Figures [file aging-12-103409-s002..pdf]

SUPPLEMENTARY FIGURES

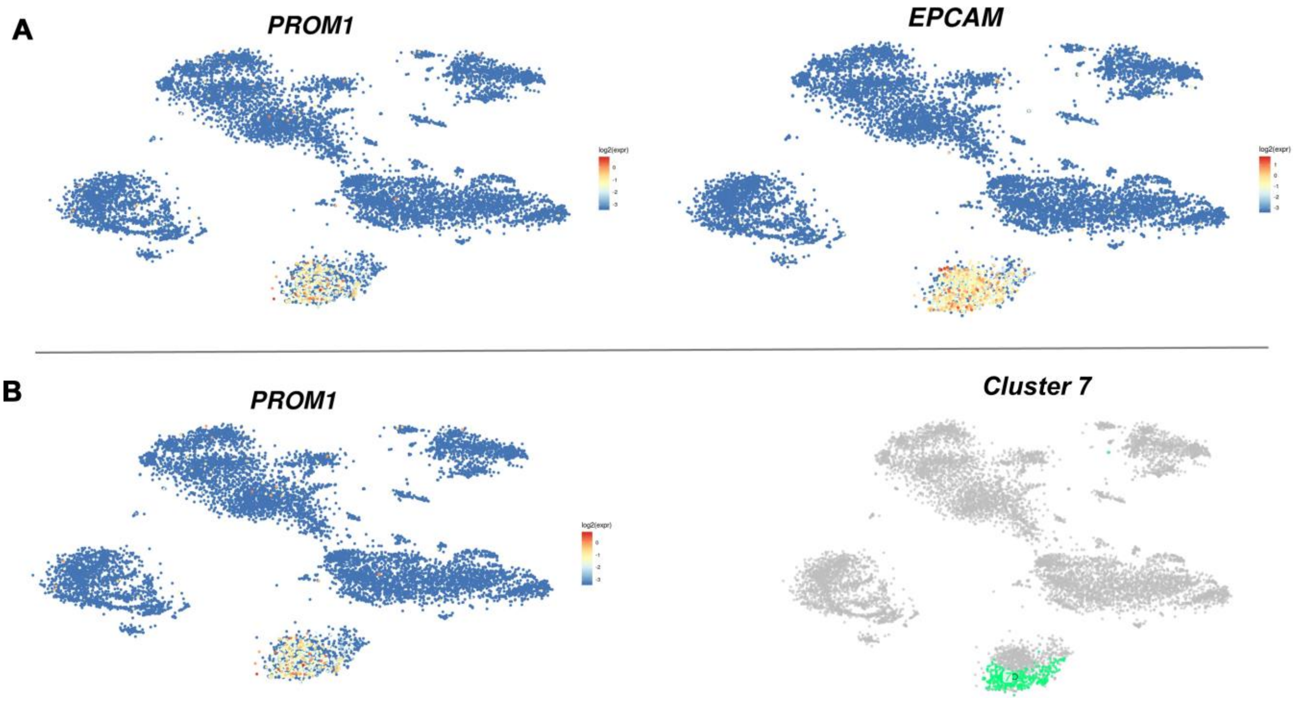

Supplementary Figure 1. The CD133 fraction is mainly expressed in endothelial cells.

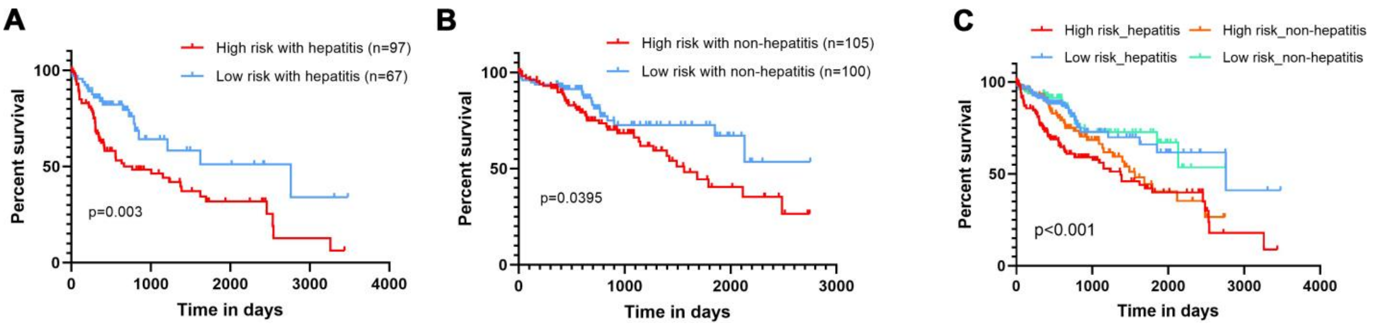

Supplementary Figure 2. Our risk model behaves well considering the inflammatory conditions of the liver.

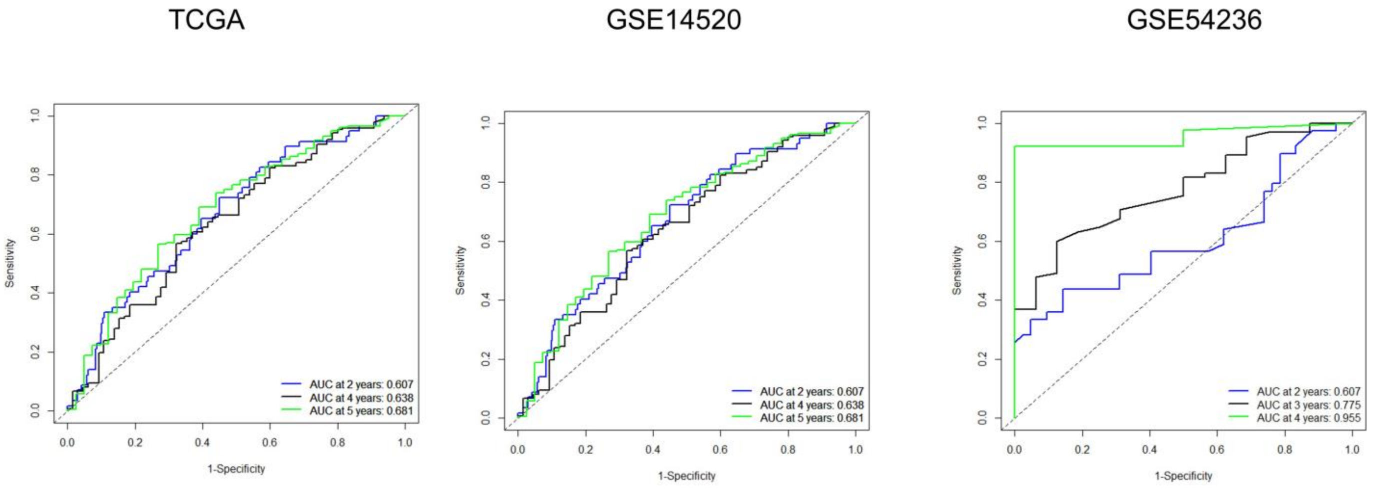

Supplementary Figure 3. Time-dependent ROC curves for the survival prediction of the prognostic model.
