# Supplementary material for: A new risk model comprising genes highly correlated with CD133 identifies different tumor-immune microenvironment subtypes impacting prognosis in hepatocellular carcinoma
Source: Aging (Albany NY). 2020 Jun 20;12(12):12234–50. doi: 10.18632/aging.103409 (PMC7343494; doi:10.18632/aging.103409)
Supplement: Supplementary Tables [file aging-12-103409-s001..pdf]

SUPPLEMENTARY TABLES

Supplementary Table 1. Significance and HR of the optimal genes associated with OS.

Gene names	Coef	HR	P value
LGALS3	0.1376	1.1476	0.009
RFX6	0.1089	1.1151	0.029
ADH6	-0.0895	0.9144	0.032
UCHL1	0.0742	1.077	0.009

*HR, Hazard ratio; OS, overall survival; Coef, relative coefficient

Supplementary Table 2. Annotation of the significant enriched GSEA datasets.

Gene sets name	Description
WOO_LIVER_CANCER_RECURRENCE_UP	Genes positively correlated with recurrence free survival in patients with hepatitis B-related (HBV) hepatocellular carcinoma (HCC);
ANASTASSIOU_MULTICANCER_INVASIVENESS_SIGNATURE	Invasiveness signature resulting from cancer cell/microenvironment interaction;
CHIANG_LIVER_CANCER_SUBCLASS_CTNNB1_DN	Top 200 marker genes down-regulated in the 'CTNNB1' subclass of hepatocellular carcinoma (HCC);
CHIANG_LIVER_CANCER_SUBCLASS_PROLIFERATION_UP	Top 200 marker genes up-regulated in the 'proliferation' subclass of hepatocellular carcinoma (HCC); characterized by increased proliferation, high levels of serum AFP, and chromosomal instability;
LU_TUMOR_VASCULATURE_UP	Genes up-regulated in endothelial cells derived from invasive ovarian cancer tissueGenes up-regulated in endothelial cells derived from invasive ovarian cancer tissue;
REACTOME_INTERLEUKIN_10_SIGNALING	Interleukin-10 signaling
GO_REGULATION_OF_MONONUCLEAR_CELL_MIGRATION	Any process that modulates the rate, frequency or extent of mononuclear cell migration.
HALLMARK_EPITHELIAL_MESENCHYMAL_TRANSITION	Genes defining epithelial-mesenchymal transition, as in wound healing, fibrosis and metastasis.
HALLMARK_INFLAMMATORY_RESPONSE	Genes defining inflammatory response
GSE7218_UNSTIM_VS_ANTIGEN_STIM_THROUGH_IGG_B_CELL_DN	Genes down-regulated in B lymphocytes: expressing IgM BCR fusion and untreated versus expressing IgMG BCR fusion and treated by anti-HEL.
GSE25123_CTRL_VS_ROSIGLITAZONE_STIM_PPARG_KO_MACROPHAGE_UP	> Genes up-regulated in bone marrow-derived macrophages with PPARG [knockout: control versus rosiglitazone
GSE36888_UNTREATED_VS_IL2_TREATED_TCELL_17H_DN	GSE36888_UNTREATED_VS_IL2_TREATED_TCELL_17H_DN

* False Discovery Rate (FDR) <0.25 and P-value of the gene sets < 0.05 was considered statistically significant.
